# Case Report: Nine-year-old with parathyroid adenoma within the piriform sinus

**DOI:** 10.3389/fendo.2023.1171052

**Published:** 2023-05-23

**Authors:** Anna Zenno, Bhavishya Ramamoorthy, Dima A. Hammoud, Martha Quezado, Martha A. Zeiger, Smita Jha

**Affiliations:** ^1^ Eunice Kennedy Shriver National Institute of Child Health and Human Development, National Institutes of Health, Bethesda, MD, United States; ^2^ Surgical Oncology Program, National Cancer Institute, National Institutes of Health, Bethesda, MD, United States; ^3^ Center for Infectious Disease Imaging, Radiology and Imaging Sciences, National Institutes of Health Clinical Center, National Institutes of Health, Bethesda, MD, United States; ^4^ National Cancer Institute, National Institutes of Health, Bethesda, MD, United States; ^5^ Metabolic Diseases Branch, National Institute of Diabetes and Digestive and Kidney Diseases, National Institutes of Health, Bethesda, MD, United States

**Keywords:** parathyroid adenomas, hypercalcemia, heritable hyperparathyroidism, hyperparathyroidism in children, parathyroid hormone

## Abstract

We present the case of a 9-year-old girl who presented with symptomatic hypercalcemia from primary hyperparathyroidism (PHPT). Laboratory results revealed elevated serum calcium 12.1 mg/dl (ref: 9.1–10.4), elevated ionized calcium 6.8 (ref: 4.5–5.6) mg/dl, phosphorus 3.8 (ref: 3.3–5.1) mg/dl, 25-OH vitamin D 20.1 (30–100) ng/ml, and elevated intact PTH 70 (15-65) pg/ml, consistent with the diagnosis of PHPT. She had persistent hyperparathyroidism after bilateral neck exploration, left thyroid lobectomy, and transcervical thymectomy. Neither inferior gland was identified. No parathyroid tissue was seen on histology. Repeat preoperative imaging identified a 7-mm × 5-mm adenoma on 4DCT not seen on ^99^Tc-sestamibi parathyroid scan. The patient then underwent a successful redo parathyroidectomy with removal of a submucosal left parathyroid adenoma at the superior aspect of the thyroid cartilage in the piriform sinus. Her biochemical work-up remains consistent with surgical cure 6 months after surgery. Herein, we also review common locations for ectopic parathyroid adenomas.

**Clinical Trial Registration:**

NCT04969926.

## Introduction

Primary hyperparathyroidism (PHPT) is a rare cause of hypercalcemia in children that is most often sporadic and due to a single parathyroid adenoma (1). The majority of affected pediatric patients are female adolescents over the age of 16 with symptomatic hypercalcemia at the time of presentation (1–3). Ectopic parathyroid adenomas are even more rare and often present as a diagnostic challenge. Among 34 published case reports of ectopic parathyroid adenoma in pediatric patients up to the age of 18 years, 15 presented with an intrathymic parathyroid adenoma (4). Other locations of ectopic adenomas were in the mediastinum, within the carotid sheath, or retropharyngeal (4). We present a 9-year-old girl with symptomatic primary hyperparathyroidism from an ectopic parathyroid adenoma located in the piriform sinus.

## Case presentation

A 9-year-old previously healthy girl (Patient-1578) presented for evaluation after experiencing 3 to 4 months of worsening nausea and vomiting, aches, and constipation. She denied bone pain, polyuria, polydipsia, confusion, or difficulty concentrating. Her only medication was ondansetron for nausea. Family history was negative for history of hyperparathyroidism or other endocrine tumors. Mid-parental height was 161.1 cm (*Z-*score −0.3). She had no history of prior neck surgery or history of neck irradiation.

### Diagnostic assessment

On examination, she had a height *Z-*score of −0.54, a weight *Z-*score of −2.37, a BMI *Z-*score of −3.35, and a blood pressure of 86/47. Her neck and abdomen were normal.

Laboratory results revealed elevated serum calcium 12.1 mg/dl (ref: 9.1–10.4), elevated ionized calcium 6.8 (ref: 4.5–5.6) mg/dl, phosphorus 3.8 (ref: 3.3–5.1) mg/dl, 25-OH vitamin D 20.1 (30–100) ng/ml, and elevated intact PTH 70 (15–65) pg/ml, consistent with the diagnosis of PHPT. Twenty-four-hour urinary calcium-to-creatinine ratio was 0.0198. Genetic testing for hyperparathyroidism was negative for variants in *AP2S1, CASR, CDC73, CDKN18, GNA11, MEN1*, and *RET* genes.

Parathyroid ultrasound showed a heterogeneous hypoechogenic lesion inferior to the right thyroid lobe, suspicious for parathyroid adenoma, but ^99^Tc-sestamibi parathyroid scan showed no scintigraphic evidence of parathyroid adenoma. In addition, ultrasound also identified sub-centimeter hypoechoic nodules in both lobes of the thyroid gland (4 × 2 × 3 mm on the right and 7 × 2 × 8 mm on the left).

### Treatment

Decision to perform bilateral neck exploration and operative localization was made in view of the patient’s ongoing symptoms. Neck ultrasound performed intraoperatively did not identify a definitive parathyroid adenoma; a left thyroid nodule, however, was noted. The superior glands were noted to be normal bilaterally and left *in situ*. However, neither of the two inferior glands could be visualized despite a complete exploration of the neck that included several intraoperative frozen biopsies determined to be thymic tissue or lymph nodes, bilateral carotid sheath exploration, retro-esophageal and retro-thyroid neck exploration, and bilateral cervical thymectomy. Additionally, because she had a left thyroid nodule and thymic limb extending into the left thyroid lobe, a left thyroid lobectomy was performed given the possibility of an intra-thyroidal parathyroid adenoma. However, the frozen section only revealed intra-thyroidal thymus. Thus, bilateral inferior parathyroid glands and the source of excess PTH remained unidentified despite complete neck exploration. Baseline PTH was 131 pg/ml, which remained flat with a final PTH of 125 pg/ml at the close of the surgery.

**Figure 1:**
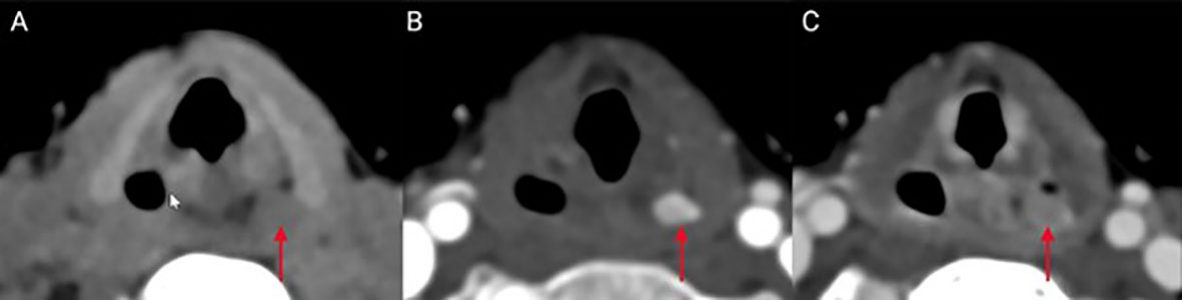
Ectopic location of the parathyroid tumor in the presented case: A left piriform sinus inconspicuous lesion (red arrow) shows similar density to adjacent muscles on CT scan images obtained prior to intravenous contrast administration **(A)** compared to avid enhancement on the arterial phase images **(B)**. Delayed (venous) imaging shows fast washout of contrast from the lesion **(C)**, consistent with diagnosis of ectopic parathyroid adenoma.

Given persistent nausea, the patient was started on potassium phosphate supplementation (500/250/250 mg at breakfast/lunch/dinner daily) with improvement of calcium to 9.1 (8.4–10.2) mg/dl and antiemetics as needed. The patient had repeat localization studies 4 months after surgery. In addition to repeat ^99^Tc-sestamibi parathyroid scan, the patient underwent a four-dimensional parathyroid CT (4DCT), which identified a candidate lesion in the hypopharynx within the piriform sinus (**Figure 1**). Retrospective comparison with ^99^Tc-sestamibi imaging did not show elevated uptake in the area of the candidate lesion identified on 4DCT. She therefore underwent a re-operative parathyroidectomy for an ectopic parathyroid adenoma within the piriform sinus. This required opening the piriform sinus in order to excise the adenoma. Baseline PTH was 157 pg/ml, which dropped to 12 pg/ml 10 min postsurgical removal. On postoperative day 9, her total corrected serum calcium was normal at 9.4 mg/dl (9.2–10.5) with phosphorus 5.6 (4.1–5.9) and PTH 19.5 pg/ml (15.0–65.0) supporting surgical remission.

### Outcome and follow-up

**Figure 2:**
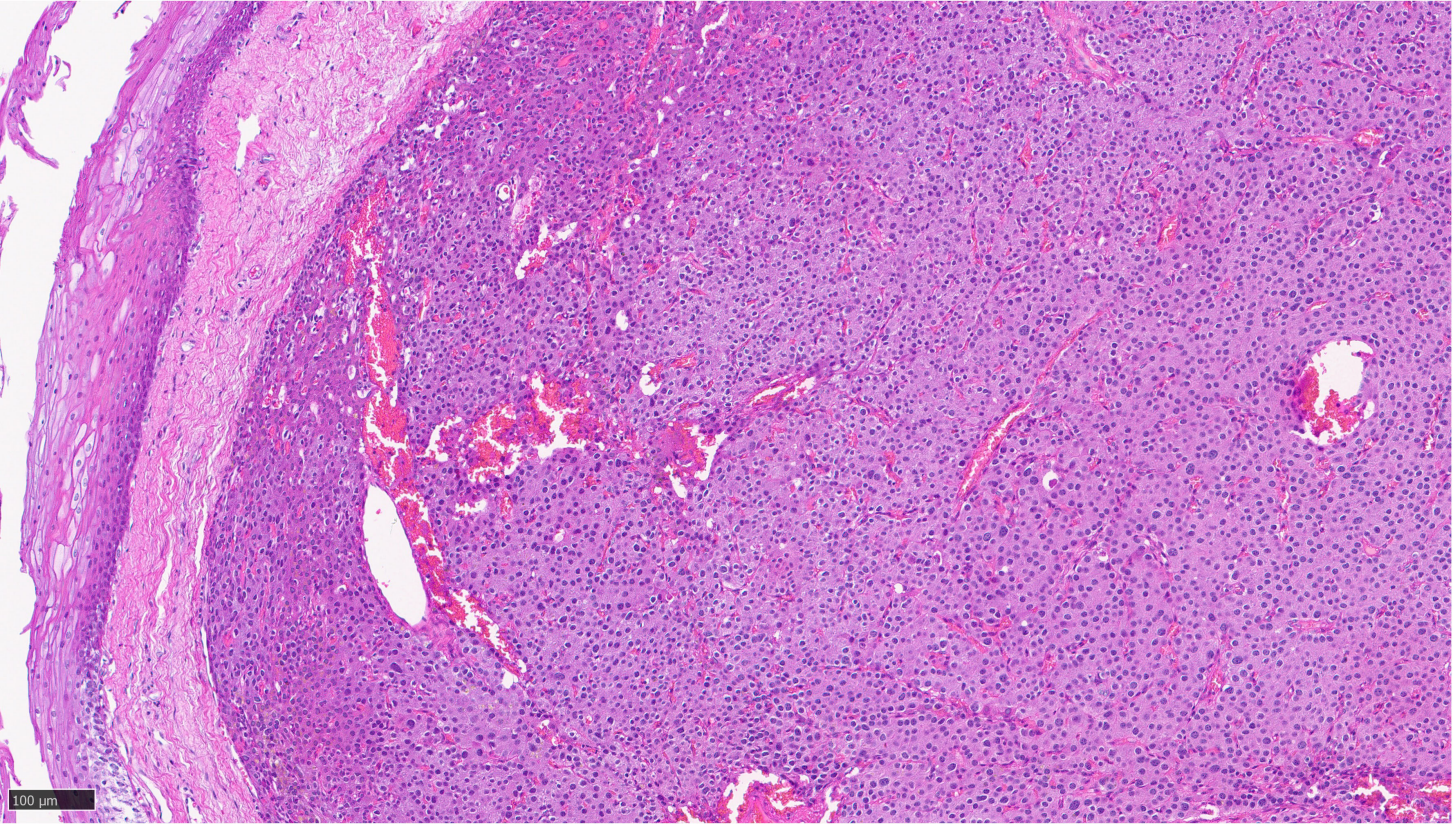
Sections of pyriform mucosa show a well-circumscribed, hypercellular parathyroid tissue composed of chief cells with a thin fibrous capsule, consistent with parathyroid adenoma.

Patient’s serum calcium, PTH, and vitamin D 6 months post-surgery was consistent with surgical cure. Surgical pathology showed a 6 × 5 × 4 mm hypercellular parathyroid tissue without any thymic remnant, weighing 500 mg (**Figure 2**).

## Discussion

PHPT is rare in children. Genetic testing should be considered in all children with PHPT, especially those with a positive family history, to exclude heritable forms. Approximately 15%–20% of parathyroid adenomas are found at ectopic locations. While the incidence of undescended parathyroid adenomas is <1%, undescended glands constitute 7% of failed cervical exploration (5). Herein, we present a rare pediatric case of PHPT from an ectopic parathyroid adenoma in the piriform sinus.

Piriform sinuses are pear-shaped recesses on either side of the laryngeal orifice. Reviewing the embryologic origins of parathyroid glands is key in understanding the piriform sinus as a possible ectopic location. Superior and inferior parathyroid glands arise from the fourth and third pharyngeal pouches, respectively, and these pharyngeal pouches are characterized by a dorsal and ventral wing (6, 7). The epithelium of the dorsal wings of the pouches differentiates into their respective parathyroid glands while the ventral wing of the third pharyngeal pouch differentiates into the thymus. The parathyroid gland primordia lose their connection to the pharyngeal wall and migrate caudally; the inferior glands are pulled down as the thymic primordia migrate caudally. Conversely, the superior glands attach to the caudally migrating thyroid gland. Piriform sinus parathyroid adenomas are specifically hypothesized to arise from failure of the superior parathyroid gland primordia to lose connection with the pharyngeal wall, whereby they may migrate to a location within the piriform sinus (8).

**Figure 3:**
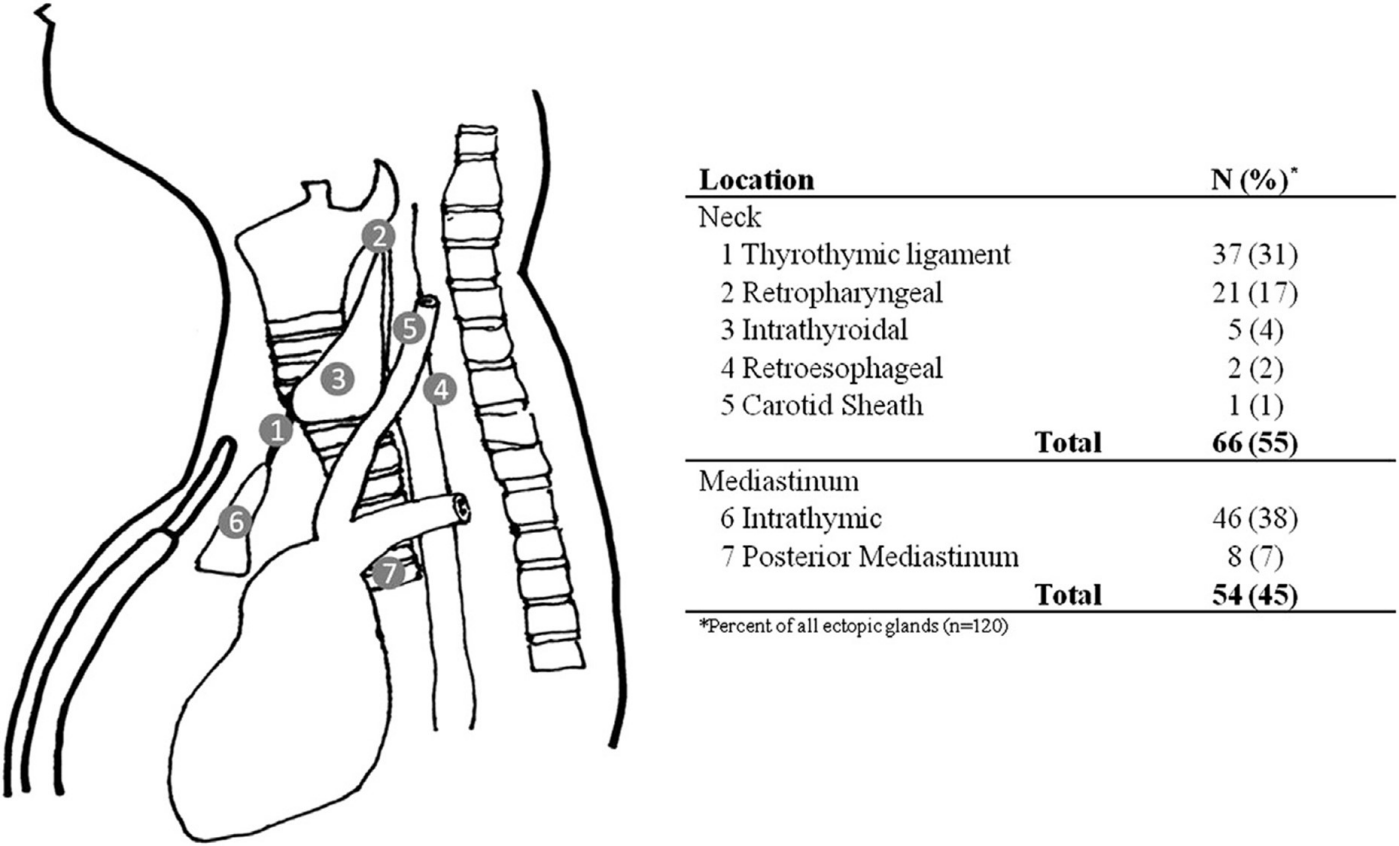
Ectopic locations of parathyroid glands and their frequency [reprinted from *Surgery*, Vol 154 Issue 3, Albuza-Cruz et al. Efficacy of localization studies and intraoperative parathormone monitoring in the surgical management of hyperfunctioning ectopic parathyroid glands, pages 453–460, Copyright (2013), with permission from Elsevier].

The term “superior” and “inferior” parathyroid gland refers to the gland’s embryologic origin and not its location in the neck (9). The inferior glands are located more anteriorly and lie anterior to the recurrent laryngeal nerve (hence, the preferred choice of remnant gland in subtotal thyroidectomy) while the superior parathyroid glands are located posterior to the recurrent laryngeal nerve. It is thought that the portion of the sinus cranial to the point of entry of the internal branch of the superior laryngeal nerve is derived from the third pouch while the sinus caudal to the point of entry is derived from the fourth pouch (5). There was no thymic tissue noted on histology of the parathyroid tumor. Since both superior glands were seen on the first operation at their expected locations, the adenoma likely represents a supernumerary gland in our patient. Supernumerary parathyroid glands are seen in 2.5%–15% of patients while <3% of patients may have only three glands (10, 11). Fourteen percent of all parathyroid adenomas are found at ectopic locations (12). While the thymus is the most frequent ectopic location of parathyroid adenomas, additional reported locations include the posterior mediastinum, thyrothymic ligament, retropharyngeal space, intra-thyroidal, retroesophageal region, or within the carotid sheath (**Figure 3**).

^99^Tc-sestamibi parathyroid scan combined with single photon emission computed tomography has the highest positive predictive value of the available imaging techniques but can be negative or misleading in patients with a gland in an ectopic position like in our case, a small gland, parathyroid hyperplasia, or multiple parathyroid adenomas (13, 14). Our group had previously reported on a series of eight patients with parathyroid adenomas in rare and unusual locations associated with various pharyngeal structures (7). ^99^Tc-sestamibi parathyroid scan successfully localized the adenoma in two out of eight patients only while an additional three out of eight patients had equivocal findings that needed confirmation on invasive localization studies like selective arteriography and selective venous sampling. Laryngoscopy has also been described as a localization procedure in patients with suspected parathyroid tumors in the upper pharynx (14–18).

4DCT combines three-dimensional imaging with the inclusion of time as the fourth dimension allowing for an evaluation of the pattern of enhancement of lesions over time (19). It is a useful imaging modality to detect ectopic glands particularly in patients with unrevealing findings on ^99^Tc-sestamibi parathyroid scan, although, unlike ultrasound, it does expose the patient to radiation, which should be minimized especially in pediatric patients.

## Learning points

- Piriform sinus is a rare ectopic location for parathyroid tumors in children.

## Data availability statement

The original contributions presented in the study are included in the article/supplementary material. Further inquiries can be directed to the corresponding author.

## Ethics statement

The studies involving human participants were reviewed and approved by NIH Intramural Program IRB. Written informed consent to participate in this study was provided by the participants’ legal guardian/next of kin. Written informed consent was obtained from the participant/patient(s) for the publication of this case report.

## Author contributions

AZ, BR, DAH and SJ collected data and presented the draft manuscript. All authors reviewed results and approved the final version.

## References

[1] Kollars J Zarroug AE van Heerden J Lteif A Stavlo P Suarez L . Primary hyperparathyroidism in pediatric patients. Pediatrics (2005) 115(4):974–80. doi: 10.1542/peds.2004-0804 15805373

[2] Hsu SC Levine MA . Primary hyperparathyroidism in children and adolescents: the johns Hopkins children's center experience 1984-2001. J Bone Miner Res (2002) 17 Suppl 2:N44–50. 12412777

[3] Lawson ML Miller SF Ellis G Filler RM Kooh SW . Primary hyperparathyroidism in a paediatric hospital. QJM (1996) 89(12):921–32. doi: 10.1093/qjmed/89.12.921 9015486

[4] Flokas ME Ganieva G Grieco A Agdere L . Ectopic parathyroid adenoma in an 11-Year-Old girl: case report and literature review. AACE Clin Case Rep (2021) 7(1):51–6. doi: 10.1016/j.aace.2020.11.013 PMC8074671 33912660

[5] Joseph MP Nadol JB Pilch BZ Goodman ML . Ectopic parathyroid tissue in the hypopharyngeal mucosa (pyriform sinus). Head Neck Surg (1982) 5(1):70–4. doi: 10.1002/hed.2890050112 7174345

[6] Benson MT Dalen K Mancuso AA Kerr HH Cacciarelli AA Mafee MF . Congenital anomalies of the branchial apparatus: embryology and pathologic anatomy. Radiographics (1992) 12(5):943–60. doi: 10.1148/radiographics.12.5.1529135 1529135

[7] Chan TJ Libutti SK McCart JA Chen C Khan A Skarulis MK . Persistent primary hyperparathyroidism caused by adenomas identified in pharyngeal or adjacent structures. World J Surg (2003) 27(6):675–9. doi: 10.1007/s00268-003-6812-3 12734681

[8] Miller DL Craig WD Haines GA . Retropharyngeal parathyroid adenoma: precise preoperative localization with CT and arterial infusion of contrast material. AJR Am J Roentgenol. (1997) 169(3):695–6. doi: 10.2214/ajr.169.3.9275880 9275880

[9] Ahn D Lee GJ Sohn JH . Ultrasonographic characteristics of pyriform sinus fistulas involving the thyroid gland. J Ultrasound Med (2018) 37(11):2631–6. doi: 10.1002/jum.14623 30099745

[10] Akerstrom G Malmaeus J Bergstrom R . Surgical anatomy of human parathyroid glands. Surgery (1984) 95(1):14–21. 6691181

[11] Carter WB Carter DL Cohn HE . Cause and current management of reoperative hyperparathyroidism. Am Surg (1993) 59(2):120–4. 8476141

[12] Dedivitis RA Guimarães AV Pontes GB . Multiple ectopic parathyroid adenomas. Sao Paulo Med J (2004) 122(1):32–4. doi: 10.1590/S1516-31802004000100008 PMC11115349 15160525

[13] Connolly MJ Lazinski D Aoki KA McLean L Torres C Dos Santos MP . Ectopic parathyroid adenoma in piriform sinus: case report and review of the literature. Ear Nose Throat J (2019) 98(1):14–7. doi: 10.1177/0145561318822933 30834784

[14] Murakami N Takeshita A Suzuki H Iizuka T Kikuchi D Matsui A . Hidden culprit of primary hyperparathyroidism. J Clin Endocrinol Metab (2012) 97(10):3410–1. doi: 10.1210/jc.2012-2190 22767637

[15] Hsieh MP Nemer JS Beylergil V Yeh R . Ectopic parathyroid adenoma of the piriform sinus on parathyroid 4D-CT and 99mTc-MIBI SPECT/CT. Clin Nucl Med (2020) 45(8):e358–e9. doi: 10.1097/RLU.0000000000003163 32558723

[16] Kim J Cubangbang M Adkins L Chia S DeKlotz TR Boyle L . Ectopic parathyroid adenoma in the pyriform sinus. Head Neck. (2017) 39(10):E110–e3. doi: 10.1002/hed.24878 28741786

[17] Muelleman T Yalamanchali S Shnayder Y . Bilateral pyriform sinus parathyroid adenomas. Ear Nose Throat J (2018) 97(3):E38–e40. 29554410

[18] Stojadinovic A Shriver CD Casler JD Gaertner EM York G Jaques DP . Endoscopic laser excision of ectopic pyriform sinus parathyroid adenoma. Arch Surg (1998) 133(1):101–3. doi: 10.1001/archsurg.133.1.101 9438768

[19] Raeymaeckers S Tosi M De Mey J . 4DCT scanning technique for primary hyperparathyroidism: a scoping review. Radiol Res Pract (2021) 2021:6614406. doi: 10.1155/2021/6614406 34094599 PMC8163538

